# A Simple Approach to Fabricate Composite Ceramic Membranes Decorated with Functionalized Carbide-Derived Carbon for Oily Wastewater Treatment

**DOI:** 10.3390/membranes12040394

**Published:** 2022-03-31

**Authors:** Umair Baig, Abdul Waheed, Basim Abussaud, Isam H. Aljundi

**Affiliations:** 1 Interdisciplinary Research Center for Membranes and Water Security, King Fahd University of Petroleum and Minerals, Dhahran 31261, Saudi Arabia; umairbaig@kfpum.edu.sa (U.B.); abdulwaheed@kfupm.edu.sa (A.W.); 2 Chemical Engineering Department, King Fahd University of Petroleum and Minerals, Dhahran 31261, Saudi Arabia; basim@kfupm.edu.sa

**Keywords:** carbide-derived carbon, membrane separation, interfacial polymerization, piperazine, terephthaloyl chloride, oil/water separation

## Abstract

Membrane-based oil–water separation has shown huge potential as a remedy to challenge oily wastewater with ease and low energy consumption compared to conventional purification techniques. A set of new composite ceramic membranes was fabricated to separate surfactant-stabilized oil/water (O/W) emulsion. Carbide-derived carbon (CDC) was functionalized by 3-aminopropyltriethoxy silane (APTES) and subsequently deposited on a ceramic alumina support and impregnated with piperazine as an additional amine. The APTES functionalized CDC-loaded membrane was then crosslinked using terephthalyol chloride (TPC). Different loadings of functionalized CDC (50 mg, 100 mg and 200 mg) were employed on the ceramic support resulting in three versions of ceramic membranes (M-50, M-100 and M-200). The fabricated membranes were thoroughly characterized by Scanning electron microscopy (SEM), X-ray diffraction (XRD), Attenuated total teflectance Fourier transform infrared (ATR-FTIR) spectroscopy, Energy dispersive x-ray spectroscopy (EDX) and elemental mapping. The highest permeate flux of 76.05 LMH (L m^−2^ h^−1^) at 1 bar using 67.5 ppm oil-in-water emulsion (as feed) was achieved by the M-50 membrane, while an oil separation efficiency of >99% was achieved by using the M-200 membrane. The tested emulsions and their respective permeates were also characterized by optical microscopy to validate the O/W separation performance of the best membrane (M-100). The effect of feed concentration and pressure on permeate flux and oil–water separation efficiency was also studied. A long-term stability test revealed that the M-100 membrane retained its performance for 720 min of continuous operation with a minor decrease in permeate flux, but the O/W separation efficiency remained intact.

## 1. Introduction

Clean water is essential for sustainable social and industrial development. Ever-growing human activities, climatic change, environmental pollution and overconsumption leave conventional water sources such as rainwater, glacier melts, rivers and lakes more and more scarce and extinct at an alarming rate [1]. The situation is even more deleterious for countries such as Saudi Arabia, where annual rainfall is below the global average [2], and hence has to meet the domestic and industrial demands for clean water through seawater desalination. Particularly, Saudi Aramco [3] consumes a huge quantity of water for its oil drilling operations, particularly to excavate oil from aging oil wells. In this process, an enormous amount of unusable produced water (PW) is generated, and the proper disposal of this oily water is a challenge for water remediation and environmental protection. Generally, oil in oily wastewater is classified into three categories: dissolved oil (<0.5 wt.%), emulsified oil (~10 wt.%) and dispersed/floated oil (~90 wt.%) [4]. The droplet size of floated oil is estimated to be >10 µm, while the size of an emulsified oil droplet is <10 µm, which is highly stabilized by surfactants. The small size of oil droplets and further stabilization by surfactants makes oil separation an extremely difficult task to accomplish [5]. Hence, the recovery and supply of clean water for perpetual industrial operations is a highly desirable and multi-challenging task.

The current approaches for disposing of, treating (dissolved air floatation, gravity separation, centrifugation and skimming) and recovering clean water from PW cannot cope with large-scale PW production and do not meet industrial water requirements [6]. The injection of waste PW into abandoned oil wells is not environmentally compatible, as the seepage of hazardous chemical contents in PW pollutes the groundwater table [7]. Additionally, the transportation or pumping of huge quantities of PW from oil drilling sites to disposal sites demands huge logistics and further increases the operational cost.

Membrane-based O/W separation technologies are increasingly used for the recovery of the PW generated from oil and gas drilling sites, as this method has many technological advantages such as lower energy consumption, ease of operation, efficient separation, a small footprint, no need for expensive chemicals, the flexibility of membrane tuning, the possibility of variable porous structure and minimal sludge generation [8,9,10,11,12]. Different types of membranes, including ceramics, polymeric and meshes, are used for O/W separation. The commonly used polymeric membranes include poly(vinylidene fluoride) (PVDF), polysulfone, poly(vinylidene fluoride-co-hexafluoropropylene) (PVDF-HFP), poly(vinyl chloride) (PVC) and polyethersulfone-based ultrafiltration membranes [13,14,15]. Such ultrafiltration membranes are prepared on a non-woven support such as polyester terephthalate through the phase inversion method [16]. During dope solution preparation, various porous additives are added to the polymer dope solutions leading to mixed matrix membranes (MMMs). The porous additives that are used in the fabrication of ultrafiltration MMMs include clays such as bentonite, conjugated microporous polymers, marshmallow-like gels, nanowires, nanomaterials such as TiO_2_, carbon nanotubes (CNTs), tungsten oxide (WO_3_) nanoparticles, Pluronic F127, SiO_2_, CaCO_3_ manganese dioxide (MnO_2_) and graphene [17,18,19,20,21]. In their recent work, Mavukkandy et al. fabricated new MMMs by incorporating WO_3_ nanoparticles into the PVDF-HFP polymeric membrane, leading to the development of a WO_3_@PVDF-HFP membrane [18]. To enhance the oil/water separation performance of WO_3_@PVDF-HFP, the membrane was coated with polydopamine resulting in 97.6% oil rejection under gravity filtration with a permeate flux of 384.3 LMH. Similarly, more MMMs were prepared by blending Pluronic F127, KCl and PVC, leading to a membrane with 92.8% oil rejection [21]. In this study [21], the effect of salt addition such as NaCl, KCl, NH_4_Cl, MgCl_2_ and CaCl_2_ was also studied, where KCl was found to be the best option.

Although polymeric MMMs are successfully used in separating O/W emulsions, ceramic membranes such as alumina- (Al_2_O_3_), silica- (SiO_2_), titania- (TiO_2_) and silicon carbide (SiC)-based ceramic membranes also have huge potential in the separation of O/W emulsions [22,23]. The obvious advantages of using ceramic membranes in O/W emulsion separation are high thermal and chemical stability, ease of tenability, increased hydrophilicity, relatively lower operating pressure and higher efficiency in the separation of O/W emulsion. Liu et al. synthesized a highly robust, biomimetic hierarchically macro-porous ceramic membrane using the self-assembly of modified Al_2_O_3_ [24]. The as-synthesized membrane showed exceptionally high efficiency regarding O/W separation, reaching up to 99.98%. Moreover, the membrane was found to be lightweight with high strength as it possessed a density of ≈1.02 g/cm^3^ and compressive strength of more than 15-fold that of ceramic membranes prepared by conventional methods.

Another extremely important feature of the membrane in the context of oil–water separation is surface wettability. Special oil and water surface wettability is introduced on the membrane surface to enhance the oil–water separation efficiency. The surfaces are categorized into wetting, non-wetting and super-wetting based on their water and oil contact angles. If the water contact angle (WCA) is less than 90°, then the surface is regarded as hydrophilic, while a surface is regarded as super-hydrophilic if WCA is less than 5°. Similarly, a hydrophobic surface possesses a WCA of more than 90°, whereas a super-hydrophobic surface has a WCA of more than 150°. The same is true for oleophilic and super-oleophilic surfaces with an oil contact angle (OCA) of less than 90° and 5°, respectively. In addition, oleophobic and super-oleophobic surfaces are surfaces with an OCA of more than 90° and 150°, respectively [25,26]. Accordingly, hydrophilic/super-hydrophilic surfaces preferably allow water to permeate or reject oil, while oleophilic/super-oleophilic surfaces allow oil to pass through while rejecting water via the membrane. Although such super-wettable surfaces have huge potential for the separation of O/W emulsion, these surfaces usually undergo fouling during the operation due to the oil wetting of the membrane surface, and this fouling leaves the subsequent oil–water separation less efficient. Therefore, various approaches are adopted to minimize the fouling of the membrane due to oil wetting. A highly promising strategy to address the challenge of membrane fouling is the development of underwater super-oleophobic membranes. An underwater superoleophobic surface allows water to pass while oil is not able to come into contact with the membrane surface, which results in the lowering of membrane fouling due to oil wetting on the membrane surface. In a work carried out by Lu et al. [27], the surface of a ceramic membrane was modified by depositing a series of metal oxides (MnO_2_, CuO, CeO_2_, Fe_2_O_3_ and TiO_2_) for the sake of understanding the impact of hydrophilicity on irreversible membrane fouling. Out of the investigated metal oxides, Fe_2_O_3_ showed considerably lower fouling during O/W emulsion separation, which was attributed to the highly hydrophilic nature of Fe_2_O_3_ as the deposition of Fe_2_O_3_ introduced a huge number of hydroxyl groups onto the membrane surface. Hence, the deposition of materials with certain salient features such as porosity, hydrophilicity and charge on ceramic membranes can be an excellent choice for separating O/W emulsion with minimum fouling.

Carbide-derived carbons (CDCs) are emerging as a new class of porous carbon materials with the unique feature of tenable pores at the sub-nanometer level. Generally, CDCs are synthesized by removing metal or semi-metal from a carbide source through various techniques such as electrochemical etching or high-temperature halogen treatment resulting in the generation of well-defined pores in the structure of CDCs [28]. Given the advantage of controllable pore size distribution, the CDCs found immense utilization in various applications such as gas separation [29,30,31], gas storage, energy storage, catalysis [32] and water desalination [33,34]. Furthermore, CDCs are also available in a variety of controllable morphologies such as monoliths, thin films, fibers, aerogels and powders. Another salient feature of CDCs is their high purity and high surface area of 2000 m^2^ g^−1^ [35]. Although CDCs are explored for their potential in numerous applications, the utilization of CDCs in oil/water separation is yet to be explored. Hence, the use of CDCs in fabricating new ceramic membranes for oil/water separation/de-emulsification has huge potential, which needs extensive studies.

In our continuous efforts [36] to explore membranes with a high separation efficiency of oil, the current study is aimed at designing and fabricating a new set of membranes for O/W emulsion separation. The membranes used in the study were fabricated using amine-functionalized CDC. The APTES functionalized CDC was deposited on a ceramic (Al_2_O_3_) support, and then piperazine, used as an external amine, was impregnated onto the CDC loaded Al_2_O_3_ support and finally interfacially polymerized using terephthaloyl chloride (TPC). The loading concentration of amine-functionalized CDC was varied during membrane fabrication. The membranes were thoroughly characterized by SEM, ATR-FTIR, EDX, elemental mapping and XRD. The membranes were applied for oil separation from surfactant stabilized O/W emulsion. The concentration of oil in feeds and permeates was determined by optical spectroscopy and a turbidity meter. Furthermore, the effect of applied transmembrane pressure and the concentration of feed on O/W emulsion separation was also studied.

## 2. Materials and Methods

### 2.1. Materials

Piperazine (PIP, ≥99%), terephthaloyl chloride (TPC, ≥99%), 3-aminopropyltrietheoxysilane (APTES, 99%), silicon carbide (SiC, 99%), sodium dodecyl sulphate (SDS, ≥99%) and triethylamine (TEA, ≥99.5%) were acquired from Sigma Aldrich (Burlington, MA, USA) and used as received. The porous alumina support was purchased from Lianyungang Highborn Technologies Co., Lianyungang, China (porosity 45%, pore size 0.5 micron). The used oil in all experiments was diesel oil obtained from a local petrol station.

### 2.2. Functionalization of CDC

**Figure 1:**
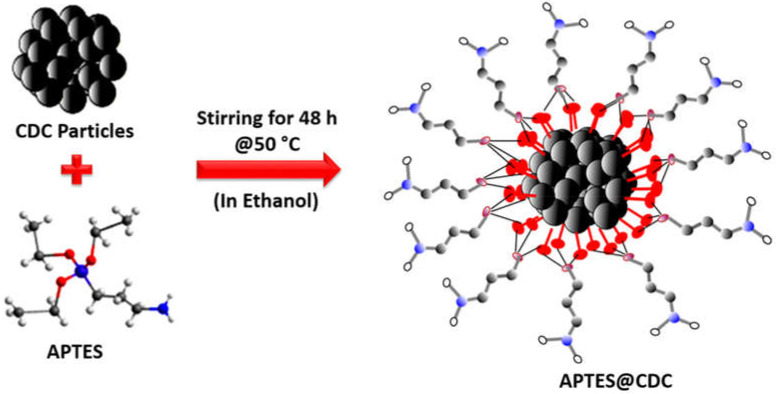
Schematic representation of the surface modification of the CDC using APTES.

The CDC was prepared as reported in our previous work [36]. The CDC was functionalized by 3-aminopropyltrietheoxysilane (APTES) using the reported procedure [37] with little modification. Briefly, the CDC (1 g) was dispersed in 100 mL ethanol (98%) using a probe sonicator and allowed to stir at room temperature for 15 min. Then, 2 mL APTES were added to the above mixture, and the reaction was allowed to proceed for 48 h at 50 °C. The reaction was stopped, and the APTES functionalized CDC was thoroughly washed with an abundant supply of ethanol and water. Finally, the APTES@CDC was dried under a vacuum before using for membrane fabrication. The possible structure of APTES functionalized CDC is shown in Figure 1.

### 2.3. Membrane Fabrication

**Figure 2:**
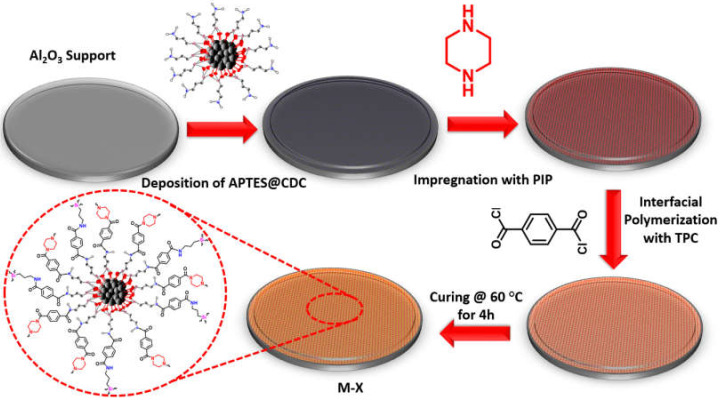
The steps involved in the fabrication of the M-X membrane.

The membrane was fabricated by depositing APTES@CDC on the ceramic support. In a typical procedure, a suspension of APTES@CDC with a concentration of 50, 100 and 200 mg/100 mL was prepared and homogenized by ultrasonication for 30 min at a frequency of 20 kHz using an ultrasound liquid processor (Sonics Materials VC-750-220; 53 Church Hill Rd, Newtown, CT 06470, USA). The alumina support (Al_2_O_3_) was mounted in a dead-end filtration cell, and 100 mL of a particular concentration of APTES@CDC were added as a feed into the dead-end cell. The APTES@CDC feed was pressurized at 1 bar through the ceramic support to deposit the APTES@CDC onto the surface of the ceramic support, and the permeate was collected as a clear solution. After physically depositing the APTES@CDC onto the surface of the ceramic support, 50 mL of 2% (wt/v) solution of PIP were passed through the membrane three times, leading to amine impregnation. The amine impregnated membrane was removed and allowed to dry at room temperature for 30 min. Afterwards, the PIP-impregnated APTES@CDC membrane was crosslinked with TPC in 0.15% (wt/v) n-hexane solution for a period of 15 min. Finally, the membrane was washed with n-hexane and subsequently cured in an oven at 60 °C for 4 h leading to a newly developed membrane which was named M-X, where X is the concentration of the APTES@CDC suspension (i.e., M-50, M-100, M-200). The various stages concerning the fabrication of the membrane are depicted in Figure 2.

### 2.4. Characterization

The functionalities present in the membrane samples were determined by exposing perfectly dried membranes to the attenuated total reflectance (ATR) mode of the Fourier transform infrared (FTIR, Nicolet iS50, Thermo Fisher Scientific, Waltham, MA, USA) in transmittance mode in the wavenumbers range of 400 to 4000 cm^−1^ [38]. The X-ray diffraction (XRD) patterns of the membranes were measured using an X-ray diffractometer (Rigaku, Tokyo, Japan) in the 2θ scan range of 10° to 90° with a 3°/min scan speed [39]. The morphological characteristics of membrane surfaces were examined by Field Emission Scanning Electron Microscopy (FESEM-Quattro, Thermo Fisher Scientific; Waltham, MA, USA) at 20 kV after gold coating with the help of a sputter coater [39]. The water contact angle (in air) and underwater oil contact angle were also measured using a drop shape analyzer (KRUSS, DSA25, Hamburg, Germany) [40].

### 2.5. Oil/Water Separation

A dead-end filtration cell (Sterlitech, Auburn, WA, USA) was used for the filtration experiments. The cell was connected to a Nitrogen gas cylinder equipped with a pressure regulator to control the applied pressure. A feed of 250 ppm oil-in-water emulsion stock solution was prepared by dispersing diesel oil and 0.05 g/L of SDS in deionized water (DI). For this, diesel oil was emulsified with water by magnetic stirring at 1500 rpm for 24 h at room temperature using SDS as an emulsifying agent. The stock solution was diluted when needed to obtain different oil concentrations. The filtration cell with the membrane (active area of the membrane = 14.2 cm^2^) was filled with 200 mL of the feed emulsion under the required applied pressure, and the permeate sample was collected and characterized after the permeation of 15–20 mL. The oil concentration of the feed and permeate was determined using a turbidity meter and optical microscopy [41,42]. Optical images of the feed and permeate samples were obtained using an optical microscope (Olympus SZX7; Tokyo, Japan). All filtration experiments were conducted in duplicate, and the average was reported.

## 3. Results and Discussion

**Figure 3:**
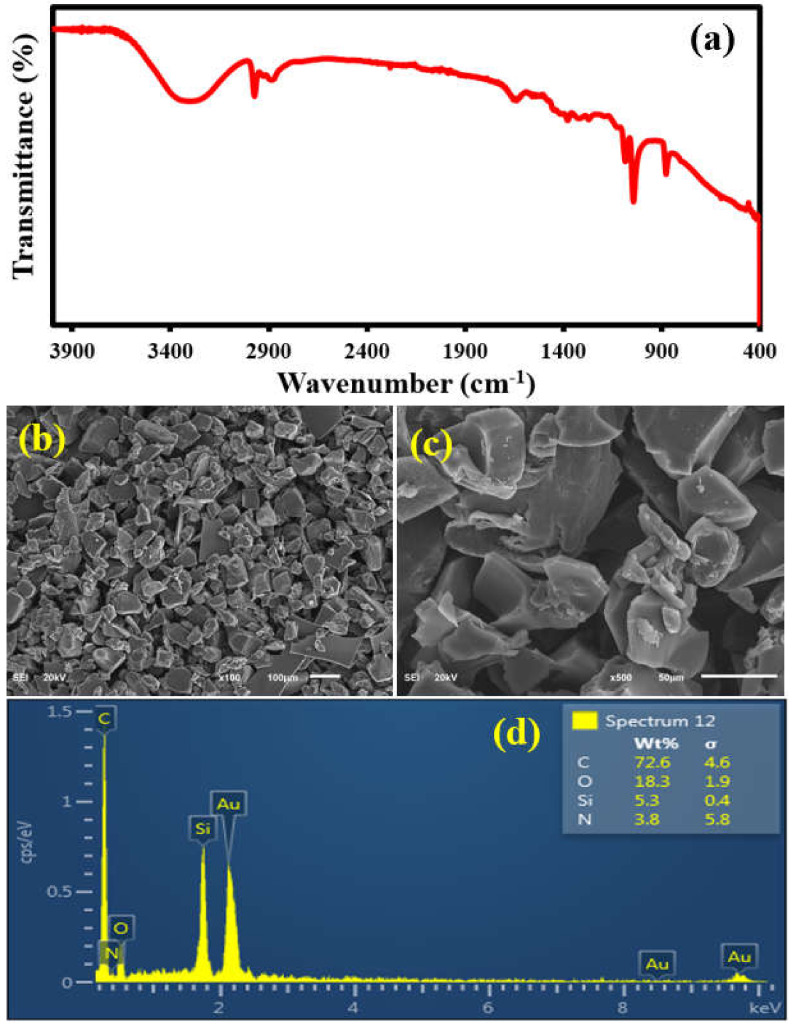
(**a**) The FTIR analysis, (**b**,**c**) the FESEM and (**d**) EDX analysis of APTES@CDC.

The spectroscopic, morphological and elemental characterizations of APTES@CDC were carried out by FTIR, FESEM and EDX analysis, as shown in Figure 3. The FTIR analysis (Figure 3a) shows the presence of characteristic peaks of APTES in the 3400–2900 cm^−1^ energy region, where both amine (-NH_2_) and aliphatic carbon chains (-CH_2_) of APTES are evident [43]. The morphological features of the APTES@CDC are shown in the images in Figure 3b,c, where irregularly shaped particles are present with a relatively smooth surface and sharp edges, as revealed by the high-magnification micrographs of APTES@CDC. Additionally, as expected, the EDX elemental analysis (Figure 3d) of APTES@CDC shows the presence of carbon (C), oxygen (O), silicon (Si) and nitrogen (N). The elemental composition of APTES@CDC comprises 72.6% C, 18.3% O, 5.3% Si and 3.8% N. The presence of all these elements is attributed to the presence of APTES in the CDC. All these observations established the successful functionalization of the CDC with APTES.

The purpose of attaching APTES to the CDC was to introduce the -NH_2_ functional group and also to fix the CDC in the APTES matrix. The addition of the -NH_2_ functional group brings about two features in the new material: (i) the covalent crosslinking of the CDC in the polyamide active (PA) layer and (ii) the fixing of the CDC onto the ceramic support to avoid its potential leaching during filtration experiments. Piperazine was also added as an additional diamine to enhance the covalent linking of the APTES@CDC in the polyamide-active layer and to tune the structure of the active layer.

**Figure 4:**
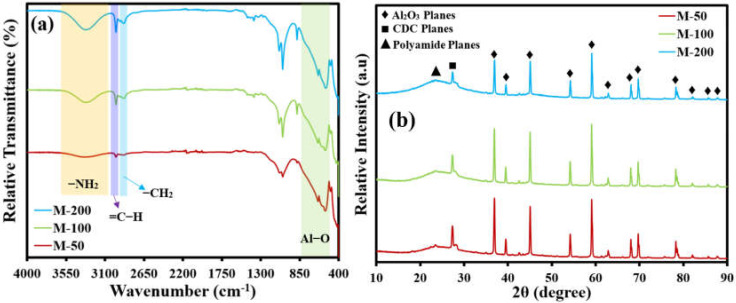
(**a**) FTIR spectra and (**b**) XRD patterns of all of the fabricated membranes.

An important characterization is the identification of the various functional groups present in the active layer of membranes. An amide identifying peak was found in the case of all membranes, which lies in a region of 3500 cm^−1^ to 3300 cm^−1^ (Figure 4a). 

An important finding was an increase in the intensity and depth of the amide peak with increasing loading of APTES@CDC in the active layer of the membrane during interfacial polymerization. This observation justifies the active contribution of the -NH_2_ function of APTES@CDC in the formation of a CDC-decorated polyamide-active layer on the ceramic support. Another highly important peak was identified in the region of 3000 cm^−1^ which is due to the aromatic =C-H bond of TPC, while small peaks in a region of 2800 cm^−1^ and 2900 cm^−1^ are due to the aliphatic -CH_2_ of ATPES. The presence of all of the anticipated characteristic peaks of reacting monomers indicates the successful fabrication of a CDC crosslinked polyamide active layer on the Al_2_O_3_ support. Moreover, a strong band present in a region of 500 cm^−1^ is due to the Al-O bond.

The X-ray diffraction (XRD) patterns of all the membranes are presented in Figure 4b. The sharp peaks spanning from 38° to 80° are attributed to the different phases of Al_2_O_3_ support, while a peak located at around 28° is due to CDC deposited on the alumina support. A hump can be seen lying in a region before the 28° peak, which can be attributed to the formation of the polyamide network, and the intensity of the hump keeps on increasing with increasing dosage loading of APTES@CDC. It also can be noticed that the peak intensities of alumina decreased with the increased deposition of APTES@CDC, which indicates the possible masking of alumina by the loaded APTES@CDC. These observations suggest the successful formation of an active layer mainly composed of CDC affixed through a crosslinked network of polyamide framework chains.

**Figure 5:**
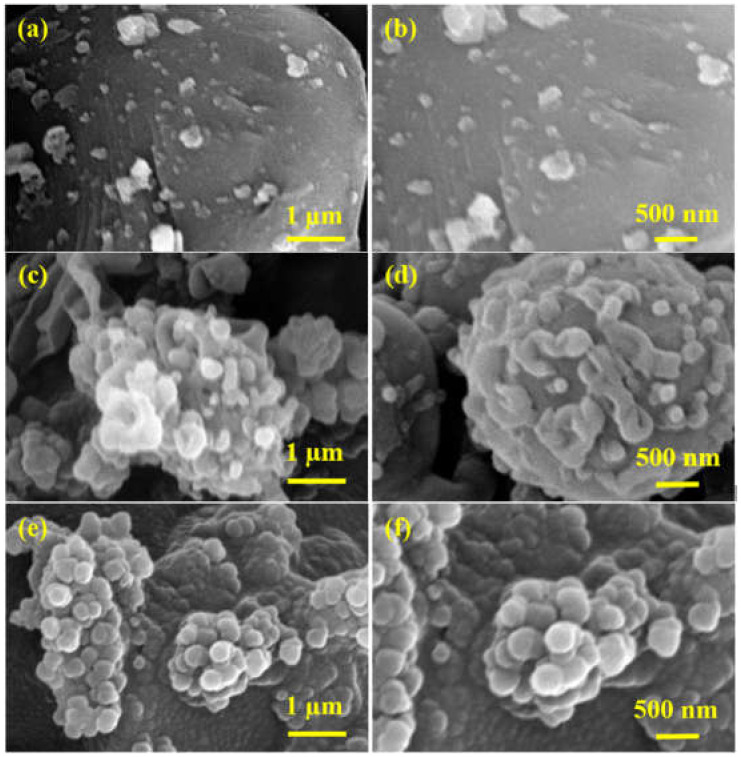
SEM micrographs of (**a**,**b**) M-50, (**c**,**d**) M-100 and (**e**,**f**) M-200 membranes.

**Figure 6:**
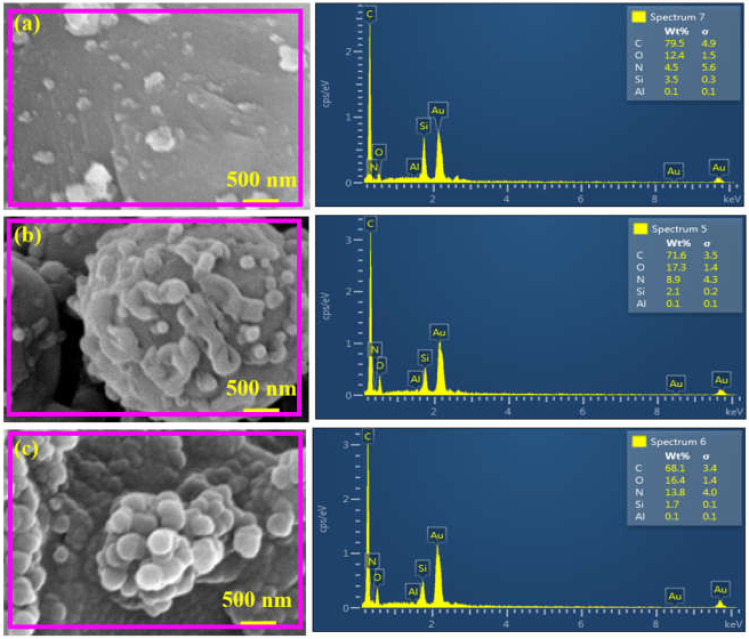
EDX analysis of (**a**) M-50, (**b**) M-100 and (**c**) M-200 membranes.

The surface morphological characterization was carried out by SEM micrographs depicted in Figure 5 and Figure S1. The micrographs show that the CDC surface is covered with globular structures, which might be attributed to the formation of a polyamide network. It is worth noting that the quantity of polymeric globules on the CDC surface increases with the increasing concentration of APTES@CDC, which indicates that more amine functional groups of APTES are taking part in a reaction with TPC during the IP reaction. Figure 5e and Figure 6f show the SEM micrographs of M-200, where it is quite evident that CDC is completely covered by a polyamide network, which could be due to the excessive loading (200 mg) of APTES@CDC during membrane fabrication.

In order to examine the elemental composition of the fabricated membranes, an EDX analysis of the membranes was carried out, and the result is shown in Figure 6, where all the anticipated elements, including C, O, N, Si and Al are present in all of the membranes. In M-50, the presence of C (79.5%) was due to CDC, ATPES and TPC, while O (12.4%) can be attributed to the TPC, APTES and Al_2_O_3_ support (Figure 6a). The nitrogen content (4.5%) was due to the -NH_2_ functional group of APTES, while Si (3.5%) and Al (0.1%) were due to the silane functional group and alumina support. Additionally, it is quite obvious that the relative mass percentage of the detected elements changed with the concentration of APTES@CDC during membrane fabrication. The change of elemental mass percent indicates a varied degree of reaction of APTES@CDC with TPC during membrane fabrication. Similar observations were also found for M-200, with N (13.8%) being more abundant than in the other two membranes (Figure 6b). Variations in the percentage of N and O (components of amide linkage; >CONH) among different membranes also highlighted a difference in the degree of crosslinking between APTES@CDC and TPC during interfacial polymerization. The O/C ratios for M-50, M-100 and M-200 were 0.15, 0.24 and 0.24, respectively. In comparison, the N/C ratios for M-50, M-100 and M-200 were 0.05, 0.12 and 0.2, respectively. The increase in O/C and N/C ratios confirms the increase in the degree of crosslinking with the increasing concentration of APTES@CDC during membrane fabrication.

**Figure 7:**
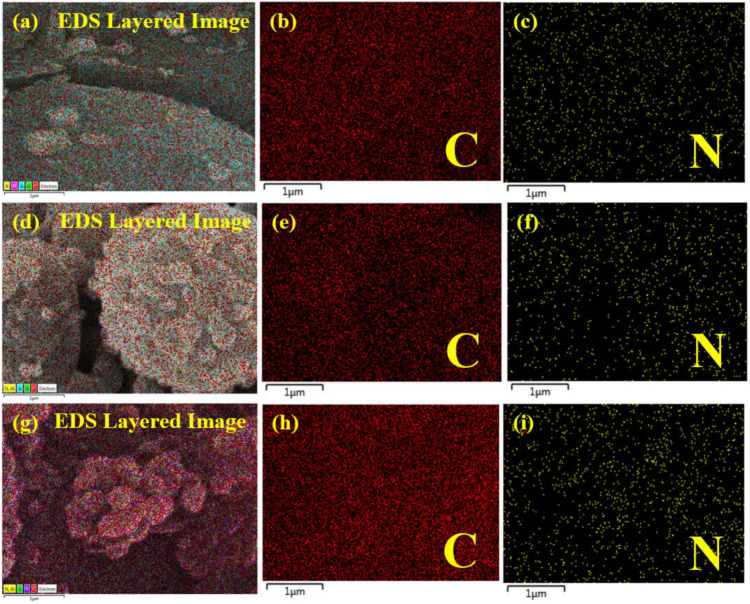
Elemental mapping of (**a**–**c**) M-50, (**d**–**f**) M-100 and (**g**–**i**) M-200 membranes.

The elemental mapping of the prepared membranes shows a uniform distribution of major elements (C and N) in the active layer of the membranes (Figure 7). It also shows a slight increase in the intensity of N with an increasing concentration of APTES@CDC.

**Figure 8:**
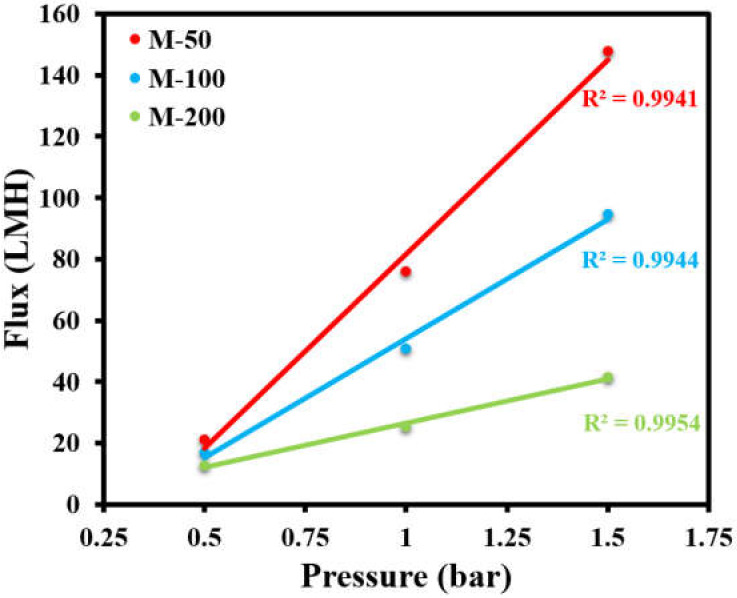
Effect of applied transmembrane pressure on permeate flux of all membranes.

After characterizing the structural properties of the fabricated membranes, the permeation properties were investigated. The permeate flux versus the transmembrane pressure was measured using 67.50 ppm oil-in-water emulsion as a feed, while the oil separation efficiency was determined using different concentrations of oil/water emulsion as feed solutions. Permeate flux was measured as a function of increasing transmembrane pressure. Even though all of the membranes showed a linear relationship between the applied pressure and permeate flux, as reflected by higher values of R^2^ > 0.99, it was noticed that the flux declined with the increasing concentration of APTES@CDC in the active layer of the membranes (Figure 8). M-50 showed a permeability of 126.76 LMH/bar, while the permeability declined to 77.74 LMH/bar in the case of M-100 and decreased even further to 28.73 LMH/bar in the case of the M-200 membrane. This observation can be attributed to the increasing degree of crosslinking during interfacial polymerization with an increasing dosage of APTES@CDC, as confirmed by EDX analysis (Figure 6). An increase in the degree of crosslinking can potentially lead to a denser active layer, which requires relatively higher transmembrane pressure for its operation.

**Figure 9:**
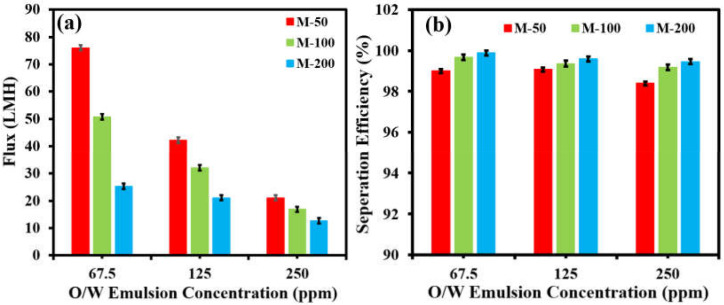
Effect of oil concentration on (**a**) permeate flux and (**b**) separation efficiency of all membranes at 1 bar.

The effect of feed-oil concentration on the performance of the membranes was also evaluated. It was observed that the permeate flux declined with increased oil concentration for all fabricated membranes (Figure 9a). The permeate flux of M-50 decreased from 76.05 LMH to 21.22 LMH as the oil concentration increased from 67.5 ppm to 250 ppm. However, it was interesting to note that the separation efficiency of all membranes remained almost constant regardless of the oil concentration (Figure 9b). M-200 showed the highest separation efficiency of 99.88%, which was followed by M-100 with an efficiency of 99.67%, while M-50 showed a separation of 99%.

**Figure 10:**
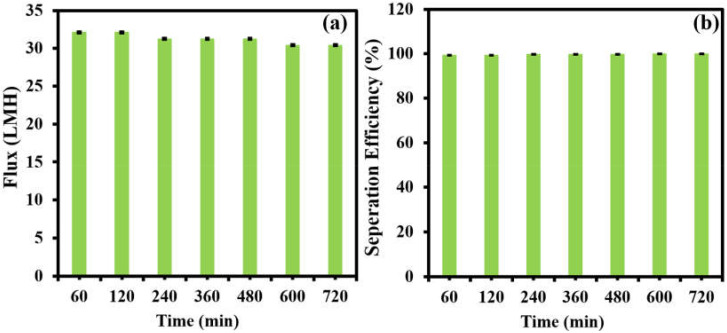
Long-term monitoring of (**a**) flux and (**b**) separation efficiency at 1 bar using oil/water emulsions at 125 ppm using an M-100 membrane.

The long-term stability of the separation membrane (M-100) was carried out using a feed concentration of 125 ppm at 1 bar. The figures of merit permeation flux and oil–water separation efficiency are characterized in Figure 10. The permeate flux (Figure 10a) and separation efficiency (Figure 10b) of M-100 were monitored over a time period of 720 min at a regular interval of 60 min. The tested membrane showed a minor decline in permeate flux from 32 LMH to 30 LMH over a period of 720 min, while the separation efficiency stayed nearly constant at 99.8% during the stability test. The long-term stability results suggest that the fabricated membranes are potentially capable of carrying out the efficient oil–water separation of oily wastewater with minimal fouling and consistent performance in terms of separation efficiency.

**Figure 11:**
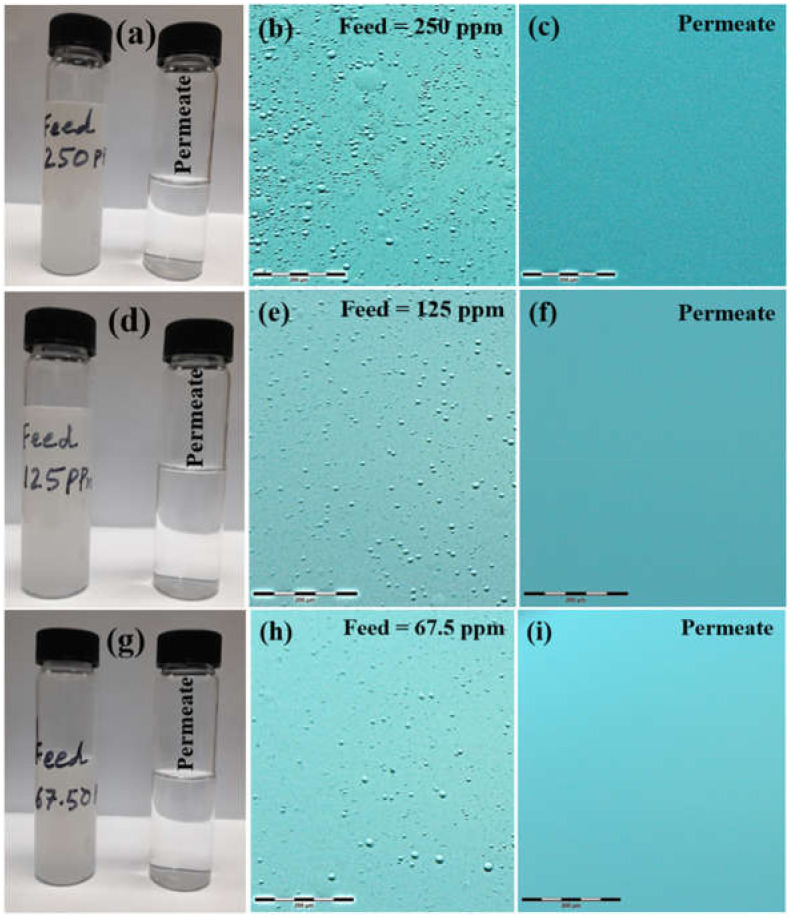
Photographs of (**a**,**d**,**g**) experimental samples of feed and permeate, optical images of (**b**,**e**,**h**) feeds and (**c**,**f**,**i**) permeates collected at 1 bar using M-100 membrane.

As visual evidence, photographs and optical images of all the tested emulsions and their respective permeates are shown in Figure 11. It was evidently clear from the sample photographs that all of the turbid feeds (67.5 ppm, 125 ppm and 250 ppm) became clear of any turbidity after permeating through the membranes (Figure 11a,d,g). Optical images of feeds (Figure 11b,e,h) and permeates (Figure 11c,f,i) showed that emulsified oil droplets were completely rejected by the membranes.

**Figure 12:**
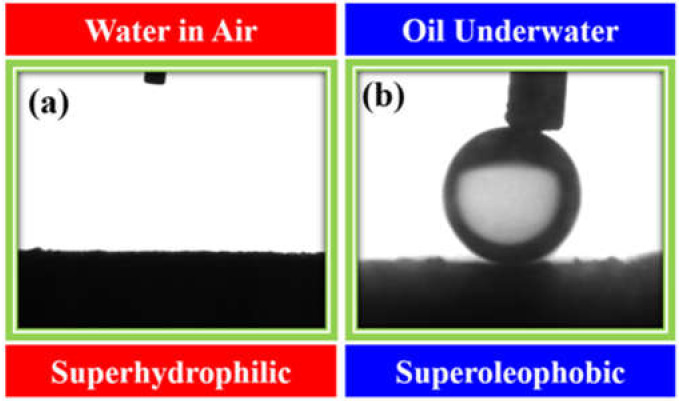
(**a**) Water contact angle in air and (**b**) underwater oil contact angle of the M-100 membrane.

As the interfacial wettability of the oil and water on the surface is the key factor for the selective permeation of oil and water through the membrane surface, we carried out the contact angle measurements of the water–surface–air interface (water in the air on the surface) and oil–surface–water interface (oil in water on the surface) using a drop shape analyzer, and the images are shown in Figure 12a,b, respectively. Clearly, the M-100 membrane is super-hydrophilic (water contact angle = 0°) in air (Figure 12a) and super-oleophobic (oil contact angle = 155°) under water (Figure 12b). The percentage of porosity of the M-100 membrane was also determined using standard protocol [44], and it was estimated to be 32.64%.

Given the huge potential of CDC decorated membranes, such membranes can be applied for various applications such as organic solvent nanofiltration and gas separation. Controlling the porosity, surface wettability, chemical compositions, morphology and other structural features of CDC, numerous novel types of membranes can be fabricated to target a specific application.

## 4. Conclusions

The current study was aimed at developing a new set of ceramic membranes for oil separation from oily wastewater. A novel approach was adopted for the fabrication of membranes where amine functionalization of CDC was carried out by APTES. Piperazine was used as an additional amine to enhance crosslinking during interfacial polymerization. The M-50, M-100 and M-200 membranes showed high permeability of 126.76, 77.74 and 28.73 LMH/bar, respectively. Among all membranes, M-200 showed the highest oil separation efficiency of >99.88%. Moreover, the long-term stability test revealed that the M-100 membrane demonstrated a stable performance for a period of 720 min. Hence, the adopted strategy during membrane fabrication was proven to be highly useful for treating O/W emulsion.

## Data Availability

Data is contained within the article.

## Supplementary Materials

The following supporting information can be downloaded at: https://www.mdpi.com/article/10.3390/membranes12040394/s1, Figure S1: SEM images of M-100 (a) and M-200 (b) membranes at low magnification.
