# Supplementary material for: A Simple Approach to Fabricate Composite Ceramic Membranes Decorated with Functionalized Carbide-Derived Carbon for Oily Wastewater Treatment
Source: Membranes (Basel). 2022 Mar 31;12(4):394. doi: 10.3390/membranes12040394 (PMC9027112; doi:10.3390/membranes12040394)
Supplement: Supplementary file 1 [file membranes-12-00394-s001.zip › membranes-1616844-supplementary.pdf]

Supplementry Information

A Simple Approach to Fabricate Composite Ceramic Membranes Decorated with Functionalized Carbide-Derived Carbon for Oily Wastewater Treatment

Umair Baig [1], Abdul Waheed [1], Basim Abussaud [2] and Isam H. Aljundi [1,2*]

[1] Interdisciplinary research center for membranes and water security, King Fahd University of Petroleum and Minerals, Dhahran 31261, Saudi Arabia; umairbaig@kfpum.edu.sa (U.B); Abdulwaheed@kfupm.edu.sa (A.W)

[2] Chemical Engineering Department, King Fahd University of Petroleum and Minerals, Dhahran 31261, Saudi Arabia; aljundi@kfupm.edu.sa (I.H.A); basim@kfupm.edu.sa (B.A)

* Correspondence: aljundi@kfupm.edu.sa; Tel.: (+966138602210)

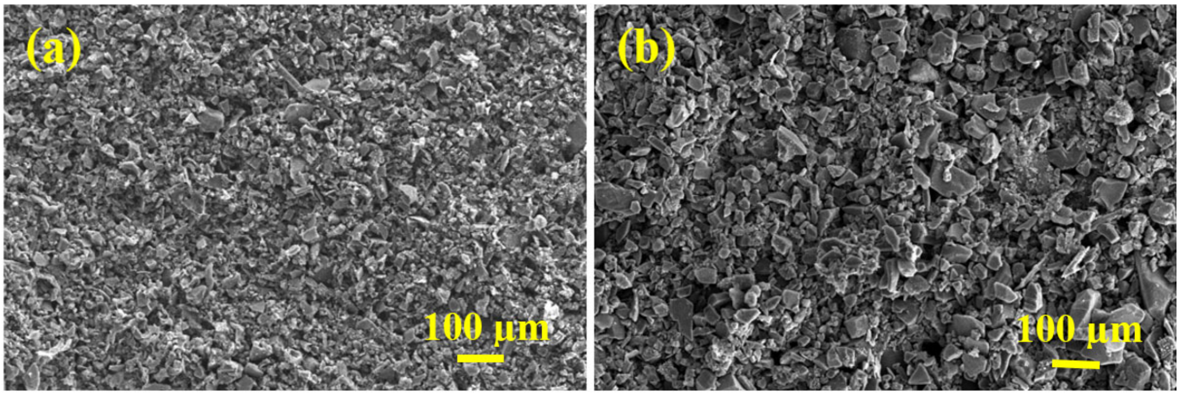

Figure S1: SEM images of M-100 (**a**) and M-200 (**b**) membranes at low magnification.
